# Norcantharidin alone or in combination with crizotinib induces autophagic cell death in hepatocellular carcinoma by repressing c-Met-mTOR signaling

**DOI:** 10.18632/oncotarget.22935

**Published:** 2017-12-04

**Authors:** Chao-Yue Sun, Ying Zhu, Xiao-Feng Li, Li-Peng Tang, Zu-Qing Su, Xie-Qi Wang, Cai-Yun Li, Hong-Mei Yang, Guang-Juan Zheng, Bing Feng

**Affiliations:** ^1^ Guangdong Provincial Hospital of Chinese Medicine, The Second Clinical College of Guangzhou University of Chinese Medicine, Guangzhou University of Chinese Medicine, Guangzhou 510120, China; ^2^ Clinical Medical College of Acupuncture and Rehabilitation, Guangzhou University of Chinese Medicine, Guangzhou Higher Education Mega Center, Guangzhou 510006, China; ^3^ School of Chinese Materia Medica, Guangzhou University of Chinese Medicine, Guangzhou Higher Education Mega Center, Guangzhou 510006, China

**Keywords:** hepatocellular carcinoma, NCTD, autophagy, c-Met, crizotinib

## Abstract

There is an urgent need for effective molecular therapies for hepatocellular carcinoma (HCC), the third-leading cause of cancer-related deaths worldwide. Norcantharidin (NCTD), a demethylated derivative of cantharidin, reportedly exhibits anticancer activity against various types of tumors, including HCC, though the mechanisms involved remain largely unknown. Here, we report that NCTD reduces viability of human MHCC-97H (97H) and HepG2 HCC cells, and induces cell death by triggering high levels of autophagy. Moreover, a significant attenuation of tumor growth was observed after NCTD treatment of HepG2 tumors *in vivo*, and this effect was enhanced by co-treatment with the c-Met inhibitor crizotinib. Interestingly, western blot analyses showed that the cytotoxic autophagy induced by NCTD correlates with a reduction in the phosphorylation status of both c-Met and m-TOR. These results suggest that cytotoxic autophagy resulting from inhibition of c-Met/mTOR signaling may be achieved in HCC by combined NCTD and crizotinib administration. Further studies to validate the therapeutic potential of this approach are warranted.

## INTRODUCTION

Hepatocellular carcinoma (HCC), the third leading cause of cancer-related death worldwide, is characterized by high metastatic and recurrence rates [1]. Despite the development of new therapeutic strategies and improved patient care, the 5-year survival rate associated with HCC remains dismal [2]. More worrisome is the fact that the death rate of HCC is increasing consistently, while the mortality associated with other malignancies is falling steadily [3]. Up to date, surgical resection or liver transplantation are still the treatment of choice for newly diagnosed cases; however, late detection renders many tumors unresectable, while shortage of organ donors often limits the number of patients that benefit from transplantation [4, 5]. Therefore, a better understanding of the mechanistic bases of HCC is urgently required to design effective molecular therapies for HCC.

The mesenchymal-epithelial transition factor (c-Met), originally identified as a fusion gene in a human osteosarcoma cell line, is a distinct disulfide-linked heterodimer that acts as a receptor tyrosine kinase for hepatocyte growth factor (HGF) [6]. Aberrant activation of c-Met signaling is responsible for the growth, progression, invasion, and poor prognosis of various types of tumors including HCC [7]. Importantly, overexpression of c-Met is frequently observed in HCC patients, and has underpinned several clinical investigations attempting to inhibit signaling through HGF/c-Met [8]. In fact, inhibition of c-Met signaling is now regarded as one of the most promising anticancer therapeutic options for the treatment of HCC [9]. Binding of HGF to c-Met induces receptor homodimerization and autophosphorylation, resulting in downstream phosphorylation of various key signaling proteins such as nuclear factor-κB (NF-κB), mitogen activated protein kinase (MAPK), and protein kinase B/ mammalian target of rapamycin (AKT/mTOR) [10–12]. Activation of the associated pathways is context-dependent and regulates critical cell functions such as proliferation, motility, and apoptosis [10].

Autophagy (“self-eating”), is a cellular catabolic process that sequesters dispensable constituents to safeguard cell activity in the face of nutrient deficiency or harsh environmental conditions [13]. Intriguingly, it has been documented that autophagy in cancer cells may contribute to either promotion or prevention of tumor growth [14]. In general, basal autophagy suppresses tumor initiation by discarding damaged mitochondria and dysfunctional proteins to keep genomic stability [15]. However, once a tumor is developed, dysfunctional autophagy often facilitates the survival of cancer cells under stress conditions [16]. Interestingly, the balance of these seemingly contradictory functions may determine tumor progression. Compelling studies have revealed that inhibition of autophagy by gene silencing or drugs could effectively kill tumor cells [17]. Some evidence, however, suggested that dysregulated, continuous autophagy could lead to cell death in HCC [18].

Norcantharidin (NCTD) is a demethylated derivative of cantharidin, a terpenoid isolated from the blister beetle (Mylabris phalerata Pall.), that was modified to enhance anti-cancer activity and reduce the intrinsic toxicity of cantharidin [19]. Many studies demonstrated that NCTD inhibits the growth of multiple tumors, including HCC [20, 21]. However, the mechanism responsible for the anti-tumor effect of NCTD on HCC has not been fully explored. In this study, we present evidence that NCTD downregulates of the c-Met and mTOR signaling pathways and induces cytotoxic autophagy in HCC cells both *in vitro* and *in vivo*, an effect exacerbated by co-treatment with the c-Met inhibitor crizotinib. Based on these data, we propose that NCTD in combination with crizotinib might be a novel and effective therapeutic approach for HCC.

## RESULTS

### NCTD inhibits proliferation and induces apoptosis in HCC cells

**Figure 1:**
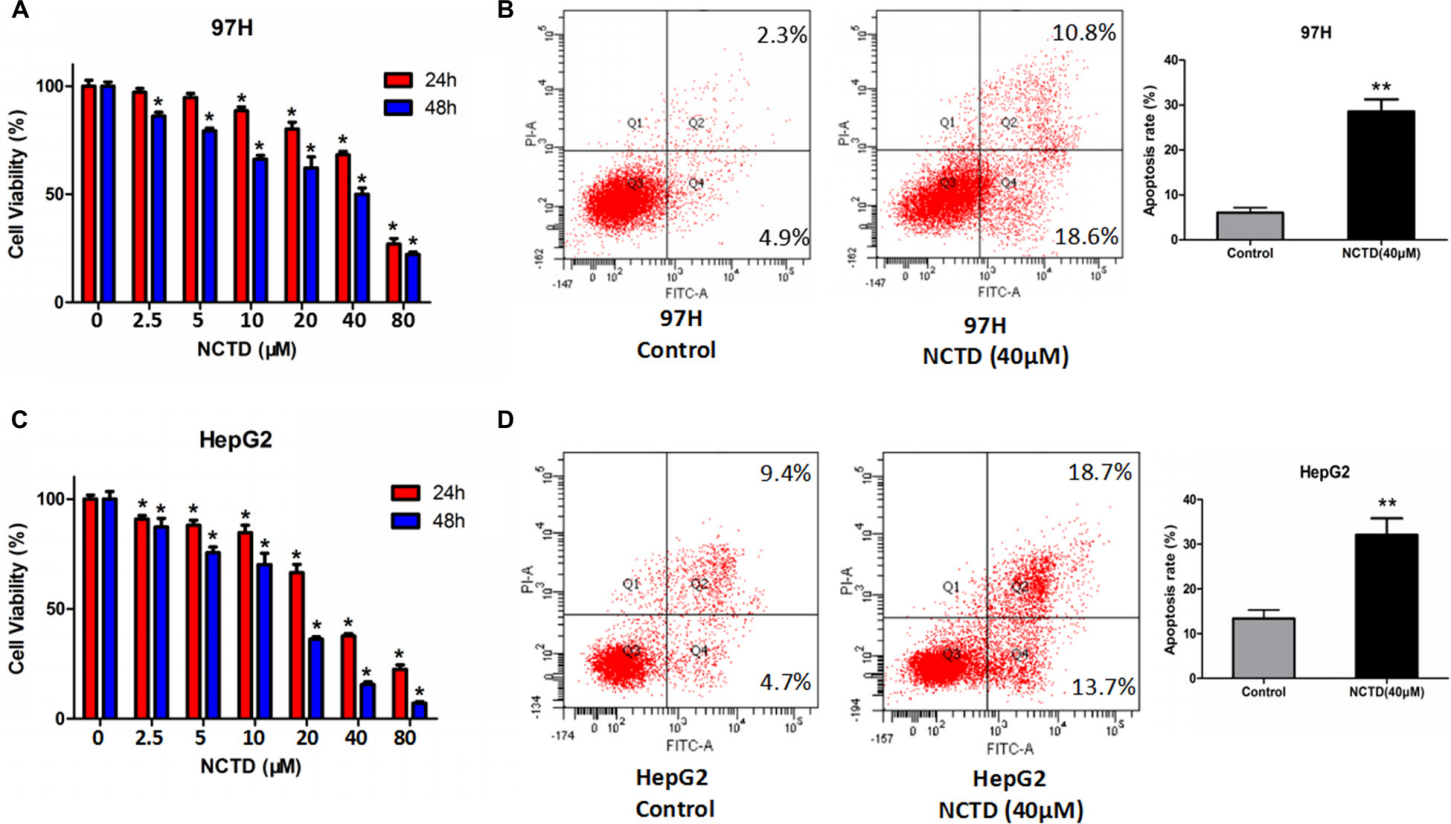
NCTD suppresses cell viability and promotes apoptosis in HCC cell lines (**A**) Cell viability assay in 97H cells treated with NCTD (0, 2.5, 5, 10, 20, 40, 80 μM) for 24 or 48 h. (**B**) Flow cytometric analysis of apoptosis in 97H cells exposed to NCTD. NCTD (40 μM) significantly increased the apoptotic rate. (**C**) Cell viability assay in HepG2 cells. (**D**) Flow cytometric analysis of apoptosis in HepG2 cells treated with 40 μM NCTD. Data are expressed as mean ± SEM. ^*^p < 0.05 and ^**^p < 0.01 compared with the control group.

To determine the growth-inhibitory actions of NCTD on HCC cell lines, MTT viability assays were performed in 97H and HepG2 cells. As shown in Figure 1A and 1C, NCTD treatment significantly inhibited the proliferation of HCC cells in a dose and time-dependent manner. After 48h treatment, the half-maximal inhibitory concentrations (IC50) of NCTD were 40.17 μM and 16.51 μM for 97H and HepG2 cells, respectively, suggesting that NCTD exerts stronger cytotoxicity on HepG2 cells. The relatively higher resistance to NCTD of 97H cells may be related to their reported higher invasive potential, compared with HepG2 cells [22]. In addition, Annexin V-FITC flow cytometric analyses showed that NCTD induced marked apoptosis in HCC cells (Figure 1B and 1D).

### NCTD induces autophagy in HCC cells

**Figure 2:**
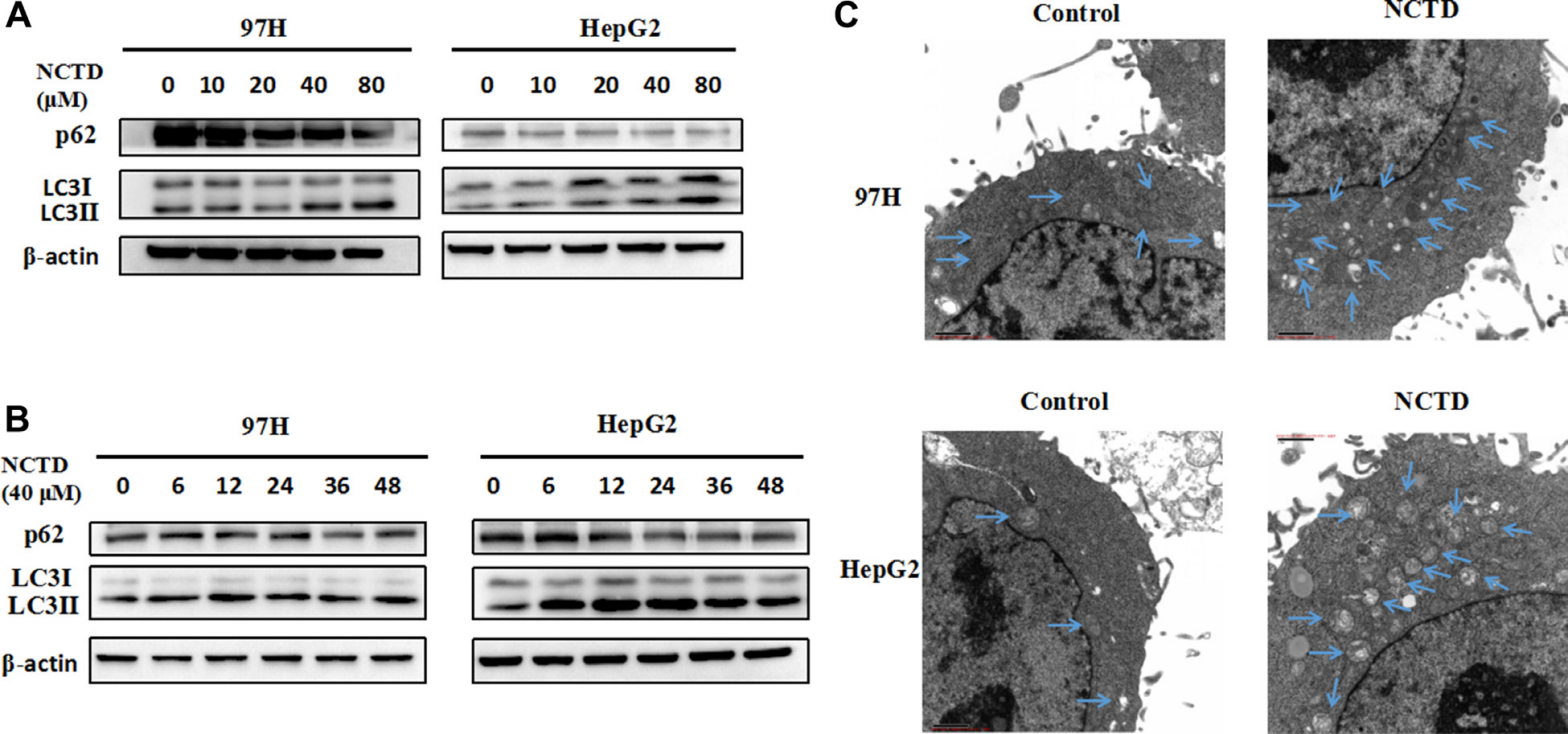
NCTD induces autophagy in HCC cells (**A**) Western blotting analysis of the autophagy-related proteins LC3 and p62 in 97H and HepG2 cells treated with NCTD (0, 10, 20, 40, 80 μM); β-actin was used as a loading control. (**B**) Western blot of LC3 and p62 in HCC cells exposed to 40 μM NCTD for 0, 6, 12, 24, 36, or 48 h. (**C**) Quantification of autophagic vacuoles in HCC cells by TEM. Cells were treated with 40 μM NCTD for 24 h.

Autophagy is widely implicated in the pathogenesis of various cancers. A previous study revealed that NCTD treatment increased autophagy in prostate cancer cells [23]. However, whether NCTD can induce autophagy in HCC cells is not known. To assess autophagy induction, we performed western blotting analyses of two autophagy-related proteins, namely microtubule-associated protein light chain 3 (LC3) and p62. LC3 is the most widely monitored autophagy-related protein [24]. During autophagy, the transition of cytosolic LC3-I to autophagosome-bound lipidated LC3-II is required for formation of the autophagosome [25]. Thus, elevations in LC3-II levels or increases in the LC3-II/LC3-I ratio are regarded as markers of autophagic activity. The adaptor protein p62, on the other hand, serves as a link between LC3 and ubiquitinated substrates and its degradation is indicative of autophagy flux [26]. HCC cells were treated with 0, 10, 20, 40, or 80 μM NCTD. Consistent with autophagy induction, NCTD treatment led to an increase in LC3-II and a decrease in p62 in both 97H and HepG2 cells (Figure 2A).

Because autophagy is a highly dynamic process, autophagic markers in NCTD-treated cells were monitored at different time points. Figure 2B shows that NCTD-induced LC3 II accumulation and p62 degradation took place at various time points, however, the maximum increase in LC3 II levels occurred 12 h after NCTD exposure. To further verify the onset of the autophagic process, transmission electron microscopy (TEM) was used to confirm the formation of autophagosomes. As shown in Figure 2C, a large number of double-membrane, autophagic vacuoles appeared in HCC cells treated with NCTD. Collectively, these results demonstrate that NCTD elicits autophagy in HCC cells.

**Figure 3:**
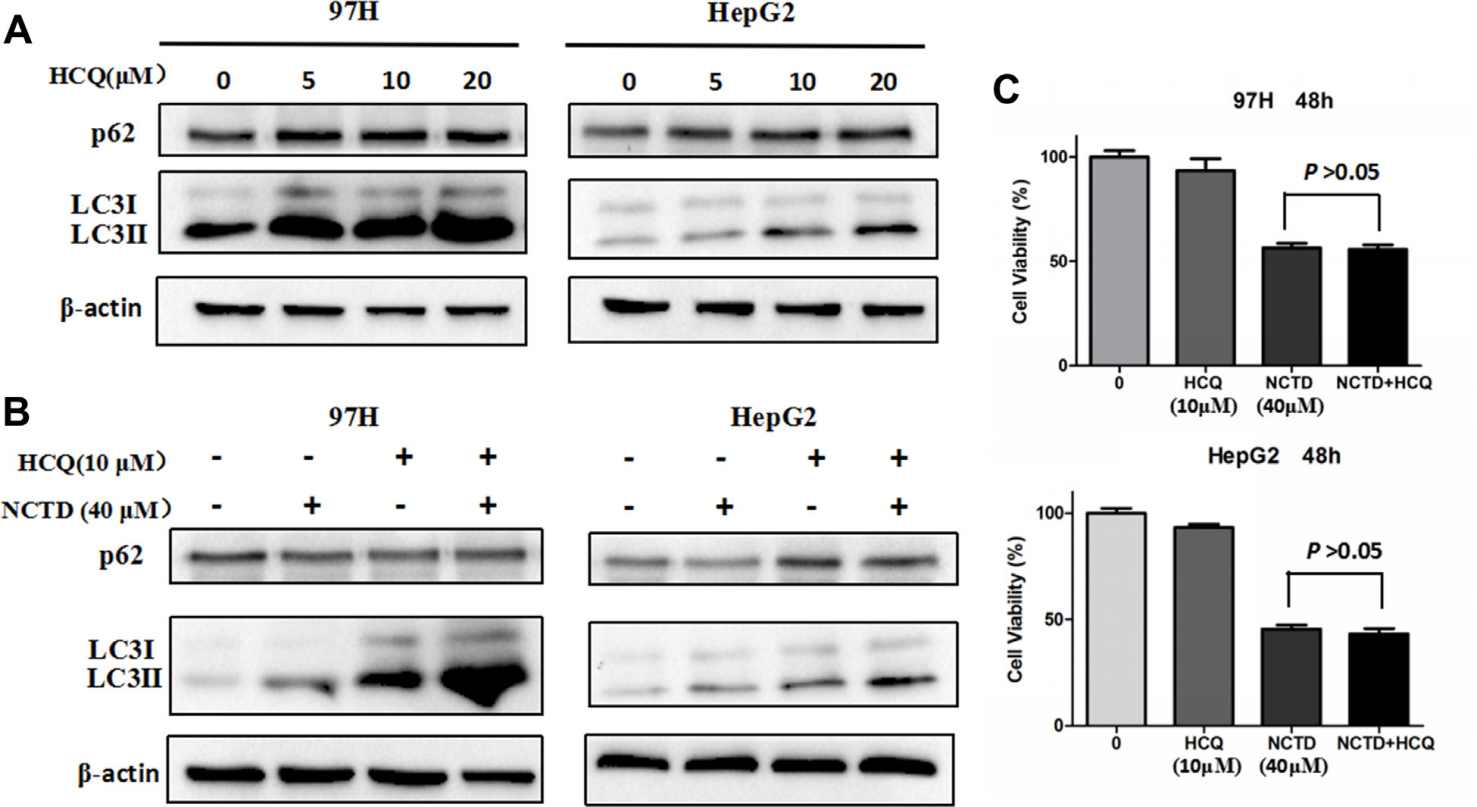
Inhibition of autophagy does not mitigate the cytotoxic effect of NCTD (**A**) Autophagy suppression by the autophagy inhibitor HCQ (0, 5, 10, 20 μM). (**B**) LC3 and p62 expression in HCC cells exposed to 40 μM NCTD, 10 μM HCQ, or both drugs combined. (**C**) Cell viability analysis in 97H and HepG2 cells; combined treatment with NCTD and HCQ did not affect proliferation. Data are expressed as mean ± SEM.

Next, we tested the effects of hydroxychloroquine (HCQ), which inhibits autophagy by impairing the lysosomal acidification of the autophagic cargo, blocking the fusion of autophagosomes with lysosomes [27]. As shown in Figure 3A, HCQ treatment strongly repressed autophagy, an effect reflected by increasing levels of both LC3 II and p62. However, in the presence of HCQ, a striking increase in LC3 II expression was still observed upon concurrent exposure to NCTD (Figure 3B). Moreover, addition of HCQ did not prevent the reduction in cell proliferation elicited by NCTD in 97H and HepG2 cells (Figure 3C), a result inconsistent with previous research [17]. These data indicate that NCTD-induced cell death could not be rescued by autophagy inhibition by HCQ.

### NCTD exposure suppresses mTOR phosphorylation in HCC cells

**Figure 4:**
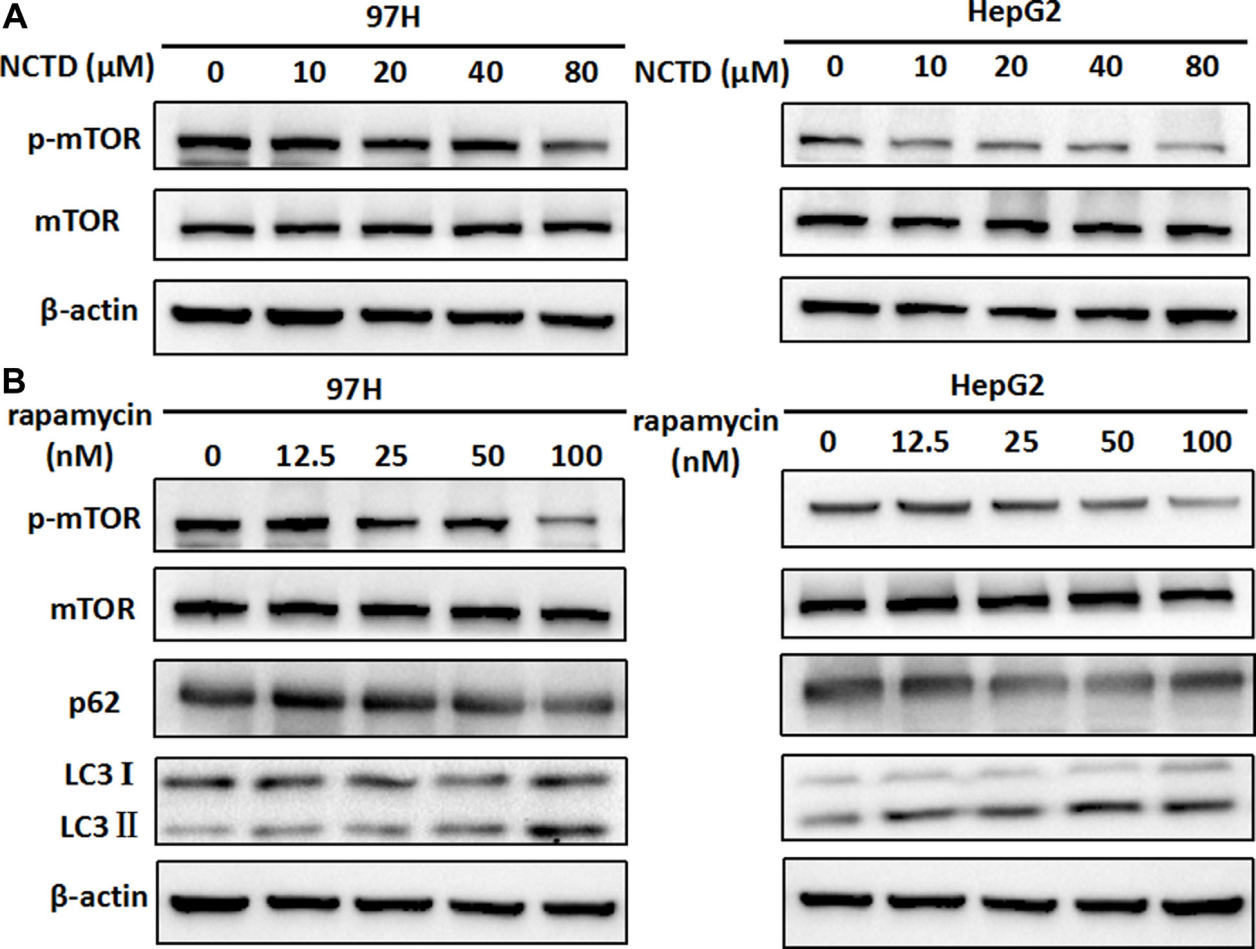
NCTD induces autophagy via suppression of the mTOR pathway (**A**) Expression of mTOR and p-mTOR in 97H and HepG2 cells treated with various concentrations of NCTD (0, 10, 20, 40, 80 μM) for 48 h. (**B**) Western blotting analysis of LC3-II expression in HCC cells; LC3-II levels increased after 48 h of rapamycin treatment, while p-mTOR expression was significantly downregulated.

Autophagy is a complex process that can be triggered or repressed by multiple signaling pathways. Among these, a key negative regulator of autophagy is represented by mTOR, a serine/threonine protein kinase that regulates gene transcription and protein synthesis according to the cell’s nutritional status [28]. Therefore, western blot experiments were conducted to assess whether mTOR inhibition was involved in the induction of autophagy mediated by NCTD in HCC cells. As shown in Figure 4A, exposure to NCTD strikingly attenuated the phosphorylation of mTOR in both 97H and HepG2 cells.

We then verified whether direct inhibition of mTOR signaling could facilitate the activation of autophagy in HCC cells by using the prototypic mTOR inhibitor rapamycin [29]. As shown in Figure 4B, no change in total mTOR was observed in rapamycin-treated 97H and HepG2 cells, compared with control. However, a modest decrease in mTOR phosphorylation, as well as accumulation of LC3 II and reduced expression of p62, indicated mTOR pathway blockade (Figure 4B). These data suggest that repression of mTOR signaling underlies the activation of autophagy by NCTD in HCC cells.

### NCTD suppresses c-Met activity in HCC cells

**Figure 5:**
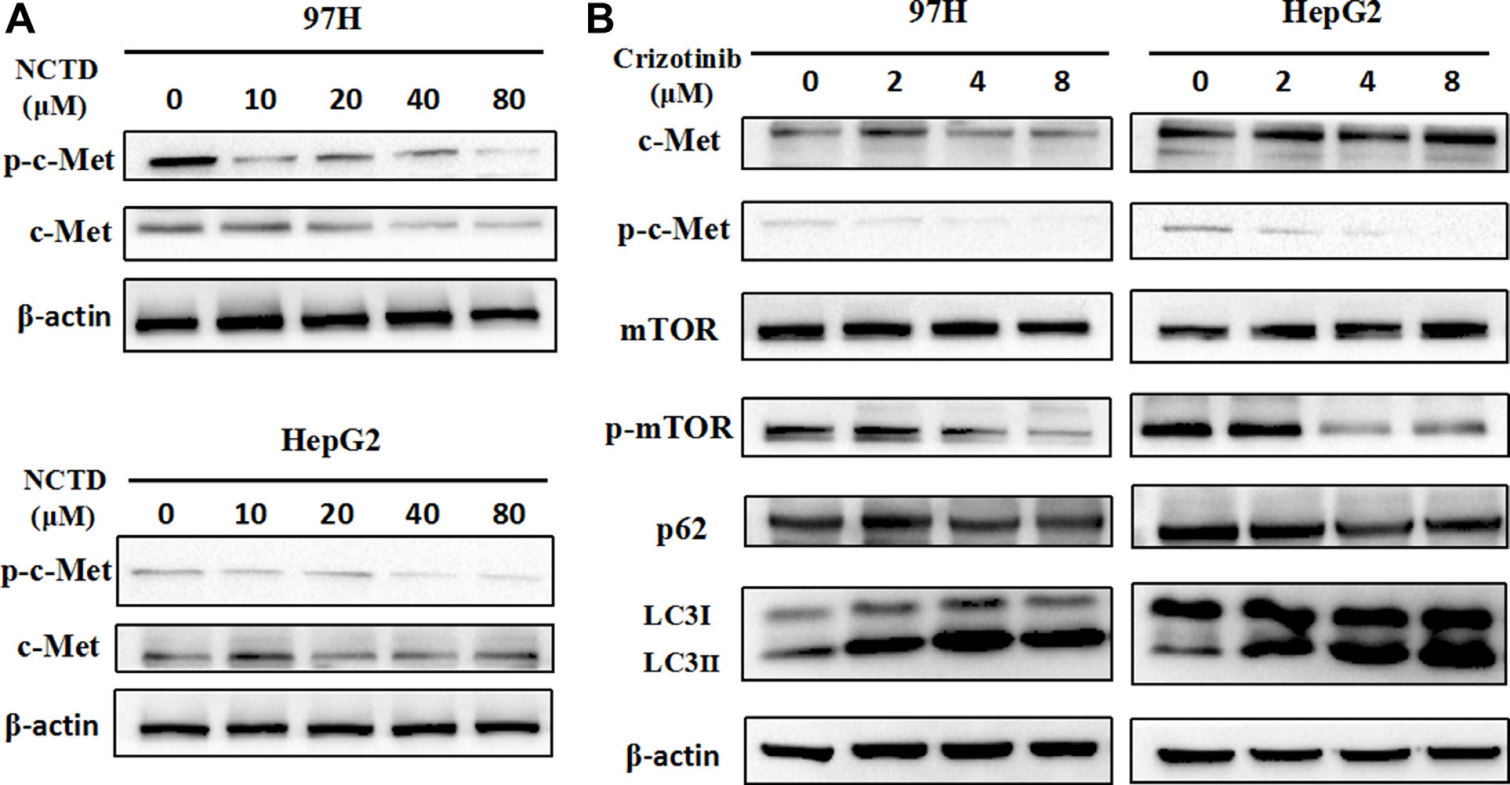
NCTD downregulates the c-Met signaling pathway (**A**) Western blot analysis of c-Met and p-Met in HHC cells treated with NCTD (0, 10, 20, 40, 80 μM) for 48 h. (**B**) Western blot analysis of c-Met, p-Met, mTOR, p-mTOR, p62, and LC3 in 97H and HepG2 cells treated with crizotinib (0, 2, 4, 8 μM).

In light of mounting evidence demonstrating that overactive c-Met signaling is a main driver of HCC progression and metastasis, we next explored the effects of NCTD on the c-Met signaling pathway by examining the expression of c-Met and phosphorylated c-Met (p-Met) by western blotting. Results showed that both c-Met and p-Met levels were markedly decreased in HCC cells treated with NCTD. In contrast, treatment with NCTD decreased p-Met levels but did not attenuate the expression of total c-Met in HepG2 cells (Figure 5A).

Given that c-Met was downregulated in HCC cells treated with NCTD, we investigated whether inhibition of c-Met would be sufficient to induce autophagy. Crizotinib (also called PF-2341066), is a multikinase inhibitor with activity against c-Met initially approved for clinical use in 2011 [30, 31]. As shown in Figure 5B, crizotinib reduced c-Met phosphorylation, while concomitantly causing a dose-dependent increase in LC3-II and a reduction in p62 expression in both 97H and HepG2 cells. Moreover, crizotinib exposure also decreased mTOR phosphorylation, suggesting that c-Met downregulation or inactivation may promote autophagy by inhibiting mTOR signaling. These results suggest that NCTD induces autophagy through inhibition of c-Met, leading to downstream repression of mTOR activation.

**Figure 6:**
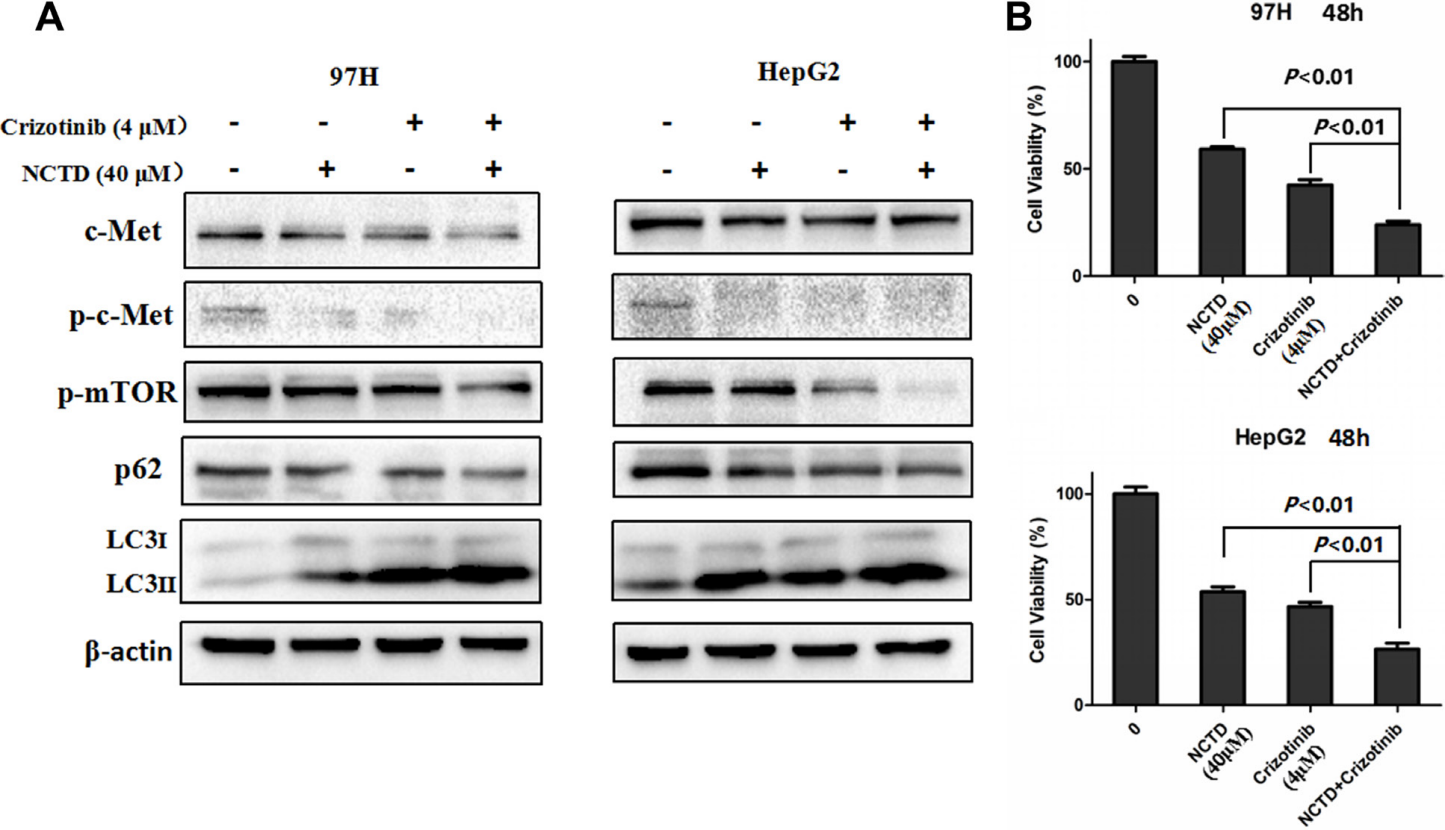
Crizotinib enhances NCTD cytotoxicity (**A**) Western blot determination of LC3 levels. NCTD (40 μM) combined with crizotinib (4 μM) significantly increased the expression of the autophagy maker LC3 through suppression of the mTOR signaling pathway. (**B**) Cell viability analysis of 97H and HepG2 cells treated with NCTD, crizotinib, or both drugs in combination. Data are expressed as mean ± SEM.

Based on the above results, we next investigated whether NCTD and crizotinib may potentiate cytotoxic autophagy in HCC cells. As expected, western blotting results demonstrated that co-treatment with crizotinib and NCTD markedly increased the percentage of LC3-II puncta and further downregulated the levels of phosphorylated mTOR (p-mTOR) (Figure 6A). More importantly, the combination of NCTD and crizotinib showed a stronger inhibitory effect on cell proliferation, compared to NCTD or crizotinib alone (Figure 6B).

### NCTD alone or in combination with crizotinib inhibits tumor growth in a xenograft mouse model

**Figure 7:**
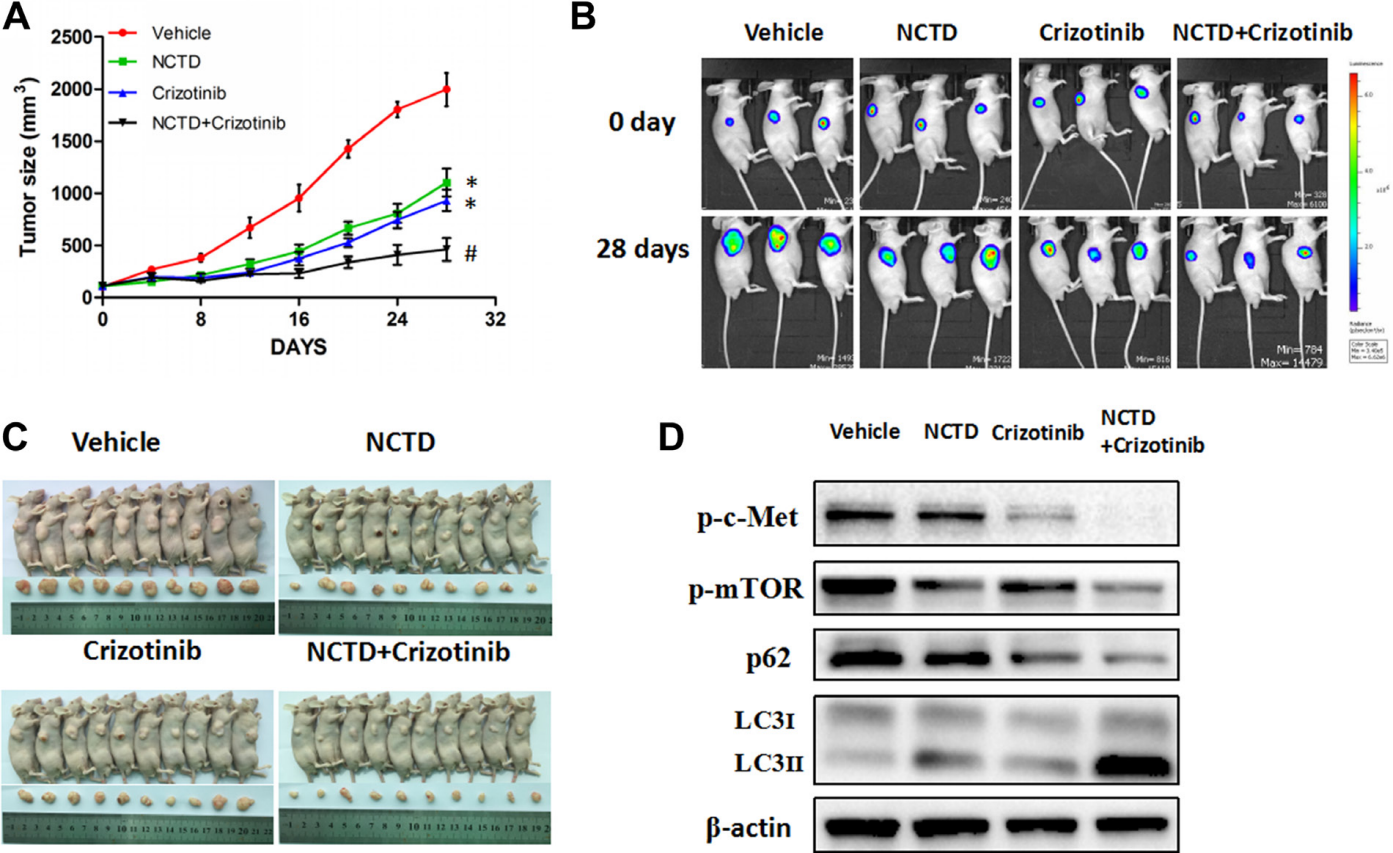
NCTD alone or combined with crizotinib inhibits tumor growth in a xenograft mouse model (**A**) Effects of NCTD, alone or in combination with crizotinib, on tumor growth. (**B**) Representative bioluminescence tumor imaging. (**C**) The images of nude mice and tumors are shown (**D**) Western blotting analysis of excised tumors. Data are expressed as mean ± SEM. ^*^p < 0.01 compared with the Vehicle group, and ^#^*p* < 0.01 compared with the NCTD or crizotinib group.

Finally, to evaluate the anti-tumor actions of NCTD *in vivo*, a xenograft model was established by injecting HepG2 cells into the flanks of nude mice. We found that administration of NCTD (30 mg/kg/day) or crizotinib (25 mg/kg/day) considerably reduced tumor size compared with vehicle-treated mice (Figure 7A). Notably, combination of NCTD and crizotinib (25 mg/kg/day) caused an even greater attenuation of tumor growth compared with NCTD alone or crizotinib (Figure 7B–7C).

To evaluate whether autophagy occurred in xenografted tumors *in vivo*, LC3 expression was measured by western blotting. In agreement with *in vitro* results, LC3-II levels were increased in tumor samples from mice treated with NCTD or NCTD plus crizotinib (Figure 7D). Moreover, attenuated expression of p-Met and p-mTOR was observed in these two groups of mice. Thus, both *in vitro* and *in vivo* data strongly suggest that NCTD alone and in combination with crizotinib inhibits tumor growth by autophagic cell death secondary to downregulation of c-Met signaling and subsequent inactivation of mTOR.

## DISCUSSION

There is an urgent need for effective molecular therapies to reduce the high mortality associated with HCC [31]. NCTD, a modified cantharidin analog, has been shown to have potent tumor suppressor activity in HCC, although the underlying molecular mechanisms remain unclear [32]. In this study, two human HCC cell lines, MHCC-97H and HepG2, were used to elucidate the mechanism mediating the anti-tumor activity of NCTD. Attesting to its potential therapeutic value, we show here that NCTD induces autophagy and causes apoptosis in HCC cells.

Autophagy, an evolutionarily conserved cellular process of self-degradation involved in the pathogenesis of HCC and other cancers, has recently aroused considerable interest in the oncological field [33]. Basal autophagy occurs in virtually all cells to meet metabolic demands, but increases when cells face starvation or encounter a variety of stress stimuli [34]. During autophagy induction, double-membraned vesicles called autophagosomes are assembled to sequester damaged or old cytoplasmic components. Subsequently, autophagosomes fuse with lysosomes, forming autolysosomes for the degradation of cellular contents [35]. The transformation of non-lipidated LC3 (LC3-I) into its lipidated form (LC3-II) with concomitant degradation of p62 represent the occurrence of autophagy [36].

The role of autophagy in cancer therapy is controversial. On the one hand, substantial work has consistently proved that the activation of autophagy confers cells resistance to various therapeutic treatments, suggesting that autophagy inhibition could strengthen the sensitivity to chemotherapeutic agents [37]. On the other hand, therapy-induced autophagy may instead lead to autophagic cell death [16]. Here, the latter notion is supported, as cytotoxic, rather than cytoprotective autophagy was induced by NCTD in HCC cells. Interestingly, the autophagy inhibitor HCQ, shown here to effectively block the autophagic process, did not attenuate under our experimental conditions NCTD-induced the death, and even caused a further upregulation of LC3 II. While this may suggest that NCTD effectively overcomes the inhibitory effect of HCQ, additional mechanisms might intervene in NCTD-induced cell death in our HCC cell models.

The mTOR pathway is the central cellular nutrient-sensing system, and prevents autophagy when the cell’s metabolic demands are met or exceeded [38]. In mammalian cells, autophagy is predominately induced by formation of ULK-Atg13-FIP200 complexes, which are direct effectors of mTORC1 [39]. Under normal physiological conditions, activated mTORC1 phosphorylates Unc-51-like kinase 1 (ULK1) and autophagy-related gene 13 (Atg13), preventing ULK1 activation by AMP-activated protein kinase (AMPK); upon stress or starvation, mTORC1 is inactivated, ULK1 becomes dephosphorylated and active, and phosphorylates in turn focal adhesion kinase family-interacting protein of 200 kDa (FIP200) as well as other targets, triggering autophagy [39]. The present investigation showed that NCTD treatment reduced mTOR phosphorylation and caused changes in the expression of LC3 and p62, two essential autophagic markers, consistent with induction of autophagy. Meanwhile, control experiments using rapamycin confirmed that inhibition of signaling through mTOR elicits autophagy in the HCC cells tested.

The overexpression of c-Met is tightly linked to clinicopathological characteristic of HCC such as prognosis, tumor grade, invasiveness and metastasis, and tumor recurrence [12]. Considering the tumor-promoting role of c-Met in HCC, many anticancer therapeutics targeting c-Met are emerging [40]. Following c-Met activation, multiple downstream signaling pathways, including NF-κB, MAPK, and phosphoinositide 3-kinase-AKT, are stimulated and contribute to tumor progression [41]. However, the potential relationship between the c-Met and mTOR pathways in HCC remains unclear.

As with mTOR, we showed here that NCTD exposure also reduced the phosphorylation status of c-Met in HCC cells. Interestingly, the c-Met inhibitor crizotinib led to a reduction not only in p-Met, but also in phospho-mTOR, and induced autophagy. In line with these individual drug effects, we demonstrate that crizotinib and NCTD enhanced autophagy in both 97H and HepG2 cells, strongly increasing cytotoxicity. Furthermore, these results were substantiated by *in vivo* experiments that showed that combination with crizotinib enhanced autophagy and caused a greater restriction in tumor growth than that observed with NCTD alone.

In summary, our findings suggest that NCTD induces autophagic cell death in HCC cells through repression of the c-Met and mTOR pathways. Notably, the combination of NCTD and crizotinib was more effective than NCTD alone in restraining both proliferation *in vitro* and tumor growth *in vivo*. These results advance our understanding of the role of autophagy in HCC, and suggest that NCTD treatment in combination with the c-Met inhibitor crizotinib might represent a promising therapeutic strategy for HCC.

## MATERIALS AND METHODS

### Chemicals and antibodies

NCTD of analytical grade was purchased from Sigma Aldrich (St. Louis, MO, USA), and dissolved in dimethylsulfoxide (DMSO) in a stock of 1 mmol/l. Hydroxychloroquine (HCQ), rapamycin, and crizotinib were obtained from Selleck Chemicals LLC (Houston, TX, USA). HCQ was dissolved in purified water, and stock solutions of rapamycin and crizotinib were prepared in DMSO. Primary antibodies against LC3, p62, c-Met, p-Met, mTOR, p-mTOR, and β-actin, as well as HRP-conjugated anti-rabbit secondary antibodies, were bought from Cell Signaling Technology (Boston, MA, USA). Fetal bovine serum (FBS) and culture medium were obtained from Invitrogen (Carlsbad, CA, USA).

### Animals

Four-to-six week-old female BALB/c nude mice were purchased from Guangdong Medical Laboratory Animal Center (Fushan, Guangdong, China). All mice were maintained in a specific pathogen-free (SPF) room, and given a standard laboratory diet and water. All the animal experimentations were approved by the Animal Care and Use Committee at Guangzhou University of Chinese Medicine, in compliance with the Guide for Care and Use of Laboratory Animals (National Institutes of Health, USA).

### Cell lines and culture

The human HCC cell lines MHCC-97H (97H) and HepG2 were obtained from Shanghai Life Sciences Research Institute Cell Resources Center. 97H cells were cultured in standard Dulbecco’s minimal essential medium (DMEM) supplemented with 10% FBS, and HepG2 cells were maintained in RPMI-1640 medium containing 10% FBS. Cells were incubated in a standard incubator with 5% CO_2_ at 37°C.

### Cellular viability assay

Cell viability was determined using the 3-(4,5-dimethylthiazol-2-yl)-2,5-diphenyltetrazolium bromide (MTT) assay. Briefly, cells were seeded on 96-well plates (4 × 10^3^ cells/well) and cultured overnight. Then the cells were exposed to various concentrations of NCTD alone, crizotinib alone, or both drugs in combination. After 24 or 48 h of treatment, 20 μL of MTT solution (5 mg/ml) was added to each well and cells were incubated for another 4 h. The media in the wells was discarded and replaced by 150 μl of DMSO, and optical density (OD) was detected at 490 nm using a multiwell spectrophotometer microplate reader (Bio-Rad, Hercules, CA, USA).

### Flow cytometry

To evaluate apoptosis, HCC cells were trypsinized, washed with PBS, and centrifuged for 5 min. After resuspension in 500 μl of binding buffer, cells were labeled with 5 μl Annexin V-FITC and 10 μl of a PI solution provided in the Annexin V-FITC/PI Apoptosis Kit (MultiSciences Biotech Co., Ltd). After a 5 min incubation at room temperature in the dark, a flow cytometer (BD Biosciences, San Jose, CA) was used to evaluate the apoptosis rate of 97H and HepG2 cells.

### Transmission electron microscopy (TEM)

TEM was used to analyze the ultrastructural features of autophagy. In brief, treated or untreated HCC cells were washed with PBS and fixed in 2.5% glutaraldehyde for 4 h at 4°C. Then the samples were post-fixed with 1.5% osmium tetroxide and dehydrated in graded ethanol. After embedment in Epon-Araldite resin, ultrathin sections (50 nm) were prepared and dyed with uranyl acetate and lead citrate. An Hitachi H-7650 (Tokyo, Japan) electron microscope was used to observe the autophagosomes at 80 kV voltage.

### Western blot

Cells or tissues were lysed in RIPA buffer, and the BCA assay was used to quantify protein concentrations. Equal amount of proteins (40 μg per lane) were resolved in 8% or 12% acrylamide gels by sodium dodecyl sulfate polyacrylamide gel electrophoresis (SDS-PAGE), and subsequently transferred onto polyvinylidene fluoride (PVDF) membranes. After blocking with 5% skimmed milk, the membrane was incubated with primary antibodies (LC3, p62, c-Met, p-c-Met, mTOR, p-mTOR, β-Actin) at 4°C overnight, followed by incubation with a secondary anti-rabbit antibody (1:5,000 dilution) for 1 h at room temperature. Protein bands were visualized by an enhanced chemiluminescence (ECL) system and measured using Image-Pro Plus 6.0. β-actin was used as a loading control.

### In vivo experiments

HepG2 cells were transfected by lentivirus to stably express luciferase (pLenti-UBC-Luc2-T2A-mKate). After transfection, cells (2 × 10^6^) were injected subcutaneously into the right flanks of the mice. One week later, when the mean diameter of the tumors reached approximately 0.5 cm, mice were randomized to either vehicle, NCTD, crizotinib, or NCTD + crizotinib. NCTD was dissolved in normal saline and orally administered daily at a dose of 30 mg/kg [42]. Crizotinib was suspended in water supplemented with 0.5% Tween 80, 30% polyethylene glycol, and 5% propylene glycol, and given daily by oral gavage at 25 mg/kg as described previously [43]. Tumor size was measured every 4 days with a caliper, and calculated following the formula: length × width^2^/2. Before treatment or after last drug administration and until day 28, tumor growth was monitored by *in vivo* bioluminescence imaging (IVIS Lumina LT Series III Pre-Clinical In Vivo Imaging System). All mice were humanely sacrificed, and the tumors were harvested for western blotting analyses.

### Statistical analysis

Statistical significance was calculated by one-way analysis of variance (ANOVA) using Statistics Package for Social Science (SPSS) software (version 17.0). Data are presented as mean ± SEM, and *p* < 0.05 was considered statistically significant.
